# Extracellular Matrix Proteins and Tumor Angiogenesis

**DOI:** 10.1155/2010/586905

**Published:** 2010-06-29

**Authors:** N. E. Campbell, L. Kellenberger, J. Greenaway, R. A. Moorehead, N. M. Linnerth-Petrik, J. Petrik

**Affiliations:** ^1^Department of Biomedical Sciences, University of Guelph, Guelph, ON, Canada N1G 2W1; ^2^CIHR Group in Matrix Biology, University of Toronto, ON, Canada M5G 1G6; ^3^Department of Pathobiology, University of Guelph, Guelph, ON, Canada N1G 2W1

## Abstract

Tumor development is a complex process that relies on interaction and communication between a number of cellular compartments. Much of the mass of a solid tumor is comprised of the stroma which is richly invested with extracellular matrix. Within this matrix are a host of matricellular proteins that regulate the expression and function of a myriad of proteins that regulate tumorigenic processes. One of the processes that is vital to tumor growth and progression is angiogenesis, or the formation of new blood vessels from preexisting vasculature. Within the extracellular matrix are structural proteins, a host of proteases, and resident pro- and antiangiogenic factors that control tumor angiogenesis in a tightly regulated fashion. This paper discusses the role that the extracellular matrix and ECM proteins play in the regulation of tumor angiogenesis.

## 1. Introduction

Conventional cancer treatments typically target the epithelial component of carcinomas, which represent a varying proportion of tumors. More recently, a paradigm shift has occurred wherein epithelial cells are being evaluated as a functional and complex system along with stromal components [1]. These stromal cells are typically recruited by tumors and include fibroblasts, endothelial cells, smooth muscle cells and immune cells. Recruitment of stromal factors not only provide a structural extracellular matrix (ECM) scaffold that provides structural support, but also generates pleiotrophic effects which contribute to tumorigenecity, thus a tumor phenotype is not only characteristic of the transformed cells, but also the ECM and stroma surrounding the cells. Stromal recruitment and alterations in the ECM result in complex communication networks between cancerous cells which may provide ideal targets for future therapies [2, 3].

The extracellular matrix provides structural support for cells within a tumor providing anchorage for cells and separating tissues, however it also acts homoestatically to mediate communication between cells and contributes survival and differentiation signals. The ECM contains a basement membrane that separates cells from the interstitial matrix. At this junction, molecular components of the ECM can be found including proteoglycan, nonproteoglycan polysaccharides, and various fibrous proteins. The carbohydrate polymers and proteins are organized in such a way that an interlocking meshwork exists and is the basic framework for the ECM. 

It is known that the ECM has structural components that neoplastic cells can exploit to create a protumor environment. Studies have found that injecting tumorigenic cells into the site of origin (orthotopically) results in a more replicable onset and progression in a variety of tumors in different mouse models [2, 3]. Others have reported the necessity of the native microenvironment in order to accurately mimic the metastatic disease [4]. Our laboratory has investigated this relationship between tumor cells and the host stroma using an orthotopic model of epithelial ovarian cancer. In this model, transformed epithelial cells are injected directly under the ovarian bursa where they can then colonize and grow. In this model, the ovarian epithelial cells interact with the ovarian stroma and result in the formation of primary serous adenocarcinoma, numerous secondary peritoneal lesions, and the formation of abdominal ascites which closely replicate the features of human epithelial ovarian cancer. The importance of the interaction between the epithelial cells and the tumor stroma is apparent as this interaction causes a reprogramming of the epithelial cells, increasing their metastatic potential; when the tumorigenic epithelial cells were injected intraperitoneally instead of orthotopically, the lack of stromal interaction resulted in the formation of small spheroids within the abdomen, as opposed to well-differentiated peritoneal lesions generated with the orthotopic model [5]. 

## 2. Tumor Angiogenesis

Investigation into the roles of the tumor stroma have established that the ECM plays an important role in tumor vascularization [6]. Cancer cells which have acquired several mutations have the ability to be: self-sufficient in growth signalling via activation of oncogenes or loss of tumor suppressor genes, insensitive to antigrowth signals, unresponsive to apoptotic events, capable of limitless replication, and tumorigenic. Although all of these neoplastic properties are necessary for tumor development, they are not sufficient to become clinically relevant cancers unless the tumor is able to recruit its own blood supply [8]. In most tumors, new blood vessels are formed through a process called angiogenesis, in which new blood vessels form from preexisting vasculature [7, 8]. Tumors require the ability to establish an angiogenic phenotype, which occurs via the angiogenic switch [9]. The angiogenic switch is regulated by a balance between pro and antiangiogenic factors and when the balance is disrupted, pathological conditions such as cancer can result [10]. 

Proangiogenic factors such as growth factors and cytokines stimulate the formation of tumor blood vessels. Two of the most critical and widely studied proangiogenic factors include vascular endothelial growth factor (VEGF) [11] and basic fibroblast growth factor (bFGF) [12, 13] (reviewed in [14]). These factors stimulate endothelial cells to produce and export various proteolytic enzymes that enable cell invasion and metastasis by degradation of the extracellular and intracellular proteins of the ECM, allowing endothelial cells to proliferate, and migrate into surrounding tissues, [15]. Under normal physiologic conditions these proteolytic enzymes are involved in wound healing and matrix remodelling. Overexpression of VEGF [16, 17] and bFGF [18–21] have been shown to promote tumorigenic properties by triggering angiogenesis. Therefore, inhibition of proangiogenic factors or upregulation of antiangiogenic factors could lead to an effective therapeutic approach. Ultimately, the angiogenic shift must favour expression of antiangiogenic factors in order for vessel regression to occur. 

## 3. Regulation of Angiogenesis by the ECM

The angiogenic process is complex and involves endothelial cell proliferation and migration, degradation of the blood vessel basement membrane and associated extracellular matrix. Following endothelial cell proliferation and early tube formation, newly formed vessels differentiate into arterioles and venules, necessary to provide blood supply to tumors [8, 22]. Remodelling of the ECM is an integral component of the angiogenic process. A variety of mechanisms have been documented about how the ECM plays a pivotal role in regulating angiogenesis (reviewed by [23]). The ECM is composed of a network of fibrous proteins and glycosaminoglycans (GAGs). GAGs are carbohydrate polymers that form proteoglycans that are involved in both keeping the EMC and surrounding cells hydrated and trapping and storing growth factors. Therefore, GAG molecules may employ a variety of regulatory effects on the accessibility of angiogenic factors [24, 25]. Release of proteolytic enzymes leading to the degradation of the ECM results in the release of ECM-bound growth factors such as VEGF ([26]; reviewed by [25]). Heparan sulfate glycosaminoglycans (HSGAGs) are a diverse family of GAGs that include the syndecans, glypicans, perlecans and agrins. Members of this group of proteins play a key role in the modulation of angiogenesis. HSGAGs that are present on the surface of endothelial cells have the ability to either inhibit or promote neovascularization by mediating signalling through VEGF receptors [27] or bFGF [28, 29]. As well, HSGAGs can also act as a binding site for antiangiogenic factor endostatin [30]. Fibrous proteins include collagen and elastin both of which are well characterized structural proteins components of the skin, connective tissue and blood vessel walls. Collagen involvement in angiogenesis has recently received a great deal of attention. Metabolic inhibition of the synthesis of type I and IV inhibits capillary formation on the CAM [31]. Data has shown that components of the ECM can have both pro and antiangiogenic effects. Proteases involved in degrading the ECM and often activated during remodelling can promote angiogenesis by stimulating migration of endothelial cells or by releasing proangiogenic growth factors [32, 33]. Angiogenesis can also be inhibited when antiangiogenic compounds are secreted from the fragments formed during proteolytic cleavage of matrix molecules [34, 35]. This paper will focus on proteases and matrix-related molecules that have been found to influence tumor angiogenesis. 

## 4. ECM Proteins Involved in Remodeling and Tumor Angiogenesis

Major ECM proteins that promote angiogenesis include collagen, laminin and fibronectin. Collagen IV and laminin are predominate proteins of the basal lamina, a 50 nm wide ECM that provides structural support for endothelial cells and creates a separation from the adjacent perivascular cells. The majority of ECM proteins mediate angiogenesis through arginine-glycine-aspartic acid (RGD) motifs which bind to integrins that mediate outside in signalling. Endothelial cells in a resting quiescent state exhibit the lowest mitotic index of cells within the body [36]. Induction of angiogenesis and remodelling of the ECM is characterized by increased permeability and cytoskeletal and cell-to-cell contact changes which results in newly formed focal contacts mediated primarily by integrins. Fibronectin is produced by both activated endothelial and smooth muscle cells, levels are augmented during angiogenesis by delivery of fibronectin from circulation by increased vascular permeability. Fibronectin contains the arginine-glycine-aspartic acid (RGD) protein motifs that bind to the integrin *α*5*β*1. This integrin receptor is markedly up regulated during angiogenesis and is over-expressed in endothelial cells in tumors. Mice genetically engineered to lack the *α*5 integrin subunit die during embryogenesis due to fail of the yolk sac vasculature to form properly [37–39]. The collagen integrin receptors (*α*1*β*1 and *α*2*β*1) also play a positive role for angiogenesis. Use of a potent and specific *α*1*β*1 inhibitor Obtustatin, is able to inhibit angiogenesis in the chick chorioallantoic membrane (CAM) assay and in the Lewis lung syngeneic model [40, 41]. Finally laminin peptides derived from the *α*1 chain mediate angiogenesis *in vitro* [41–43]. Receptors involved in laminins proangiogenic properties have not been fully elucidated, *α*6*β*1 receptor may play important role in tube formation [44].

Proteolytic activity of the ECM facilitates degradation of the basement membrane, matrix remodelling, and cell migration and invasion, all of which are essential for angiogenesis. In order for angiogenesis to occur, activation of proteases is essential. However, aberrantly excessive degradation of the ECM does not permit developing vessels [32]. Therefore, in a similar fashion to the regulation of angiogenic processes by angiogenesis by pro and antiangiogenic factors, activation of proteolytic enzymes of the ECM is also tightly regulated. There are two main classes of enzymes that have been studied for their abilities to degrade and remodel the ECM: the plasminogen activator (PA)/plasmin system and matrix metalloproteinases (MMPs), which have been reviewed for their roles in angiogenesis [45]. To date, a number of MMPs have been shown to degrade the vascular basement membrane and matrix in order to permit vascular sprouting. The activity of these proteases is regulated by endogenous tissue inhibitors of metalloproteinases (TIMPs) which generally have antiangiogenic properties.

MMPs are a group of zinc-dependent proteases that are involved in the degradation and remodelling of the ECM in order for processes such as angiogenesis to occur. The MMP family consists of over 20 proteases and many of them have been implicated in tumorigenesis [32]. Those that have been reported to have proangiogenic actions are described below. By degrading the matrix, MMPs not only provide physical space within the matrix for migration, but also provide proliferation and differentiation signals to endothelial cells by releasing cryptic sites on ECM proteins and soluble growth factors. The involvement of MMPs in angiogenesis has been supported through the use of knockout mice. Studies involving knockout mice of the gelatinase type MMPs (MMP-2 and MMP-9) have shown tumor angiogenesis [46]. Researchers have subsequently shown that MMP-2 promotes an angiogenic phenotype, while suppression of the protease inhibited angiogenesis [47]. MMPs cleave ECM bound growth factors including proangiogenic factors [48]. Various MMPs have been found to cleave heparin bound growth factors such as VEGF and bFGF, releasing soluble forms which then exert proangiogenic actions and stimulate the formation of new blood vessels [49]. In particular, it was reported that MMP-9 stimulates the production of the proangiogenic growth factor VEGF [50, 51]. Other members of the MMP family have also been shown to enhance the effectiveness of proangiogenic growth factors. Membrane bound MMPs also mediate proangiogenic effects. Corneal pocket implantation assays revealed that MT1-MMP can potentiate the neovascularization effects of basic fibroblast growth factor (bFGF) [52]. When cells that do not normally express MT1-MMP were transfected with the matrix protease, angiogenesis was stimulated and *in vivo*, neovascularization was associated with an increase in expression of VEGF [53]. In a xenograft model of Glioma, cells that overexpressed MT1-MMP were capable of remodelling a matrix *in vitro* and had increased levels of angiogenesis in vivo. Consistent with other studies, these changes in angiogenesis were correlated with an increase in VEGF [54]. This is maintained during situations in which MMPs are decreased, ultimately resulting in a reduction in the levels of proangiogenic growth factors [55]. Stromal recruitment of fibroblasts and immune cells such as macrophages can also modulate MMP remodelling of the stroma altering the signalling that ultimately results in increases and decreases of angiogenesis. Recently loss of PTEN signalling in stromal fibroblasts results in the induction of ECM remodelling by the increase of the transcriptional factor Ets2 which is an upstream target of MMP9 [56]. Protease mediated cleavage of the ECM also results in the release of cryptic antiangiogenic factors. Cleavage of basement membrane proteins, collagen XVIII and the *α*1, *α*2 and *α*3 chains of collagen IV release the angiogenic inhibitors endostatin, arrestin, canstatin and tumstatin, respectively [57]. Endostatin once release from mature collagen XVIII binds to cell surface proteoglycans, VEGFR-2 and the *α*5*β*1 integrin to inhibit angiogenesis *in vitro* and *in vivo* [57]. Due to the longer half-life on these endogenous inhibitors of angiogenesis, it has been hypothesized that they accumulate in the serum of patients with larger primary neoplasia and inhibit angiogenesis at distal sites and limit the growth of metastatic foci until resection of the primary lesion [58].

As mentioned, remodelling of the ECM is a tightly regulated process. The inhibitory influence that the TIMPs have on MMP expression and function therefore is an important regulator of matrix degradation. Overexpression of TIMP-1 has shown to suppress tumorigenesis, in part due to its effects on the tumor vasculature. Immunostaining revealed that mice that overexpressed the endogenous TIMP-1 had significantly reduced tumor vessel density compared to controls (REF). *In vitro* treatment with TIMP-1 showed that tube formation was altered despite no significant changes in endothelial cell proliferation. Decreased expression of MMP-2 and MMP-9 in the tumors of TIMP-1 transgenic mice but not in the *in vitro* experiments, suggest that inhibition of the matrix degradation is not a direct effect on MMPs, but may require the presence of a reactive stroma [59]. *In vitro*, TIMP-2 decreases proliferation of endothelial cells and inhibits angiogenesis *in vivo* [60, 61]. Other *in vivo* experiments involving immunohistochemical analysis of tumors overexpressing TIMP-2 reported a decrease in microvessel density compared to controls [62]. Although the mechanisms by which protease inhibitors such as TIMP-2 inhibit angiogenesis are not well understood, it is thought to be the result of a decrease in proangiogenic factors such as VEGF [63] and bFGF [64]. TIMP-3 has also shown to decrease angiogenesis, particularly through inhibition of endothelial tube formation and disaggregation of endothelial cells [65–67]. Support for TIMP-3 as a therapeutic comes from studies involving animals that were deficient in the protease inhibitor exhibited an increased angiogenic phenotype [68]. TIMPs have been reported to not only influence the vasculature, but in some cases, exert their antiangiogenic properties through an MMP-independent mechanism [69–71]. In 2003, Fernández et al. characterized the antiangiogenic domains of TIMP-2, a protease inhibitor which decreases endothelial cell proliferation. In this study, they found that both terminal domains of the protein were capable of inhibiting angiogenesis. It was also noted that only the domain which does not function with MMPs was able to inhibit mitogen-driven angiogenesis. This can be interpreted that therapeutics that solely target MMPs to inhibit angiogenesis might not be as effective as TIMPs [72]. 

Another group of ECM proteins which have recently been studied for their role in tumor angiogenesis are a disintegrin and metalloproteinases (ADAMs) and a disintegrin and metalloproteinase with thrombospondin motifs (ADAMTs). Besides the addition of thrombospondin (TSP) motifs on these proteases, the ADAMs are associated with the membrane while the ADAMTs are secreted [73]. These proteins belong to a similar family as MMPs and many of them have been found to regulate angiogenesis directly or through expression of MMPs. ADAM-17 has been reported to play a role in angiogenesis as was evident from *in vitro* experiments on endothelial cells. Inhibiting ADAM-17 not only altered morphology of the endothelial cells but it also decreased proliferation, leaving apoptosis unchanged. In terms of elucidating how ADAM-17 influences angiogenesis, this was determined to be the result of MMP-2 activation via VEGF. The involvement of ADAM-17 was confirmed when endothelial cells lacking the protein did not have an increase in MMP-2 following VEGF treatment [74]. Evaluation of the first thrombospondin repeat (TSR1) in ADAMTS5 revealed inhibition of endothelial tube formation and proliferation [75]. Unlike ADAM-17, ADAMTS5 induced endothelial cell death even in the presence of VEGF, a potent proangiogenic and growth promoting factor. ADAMTS1 and ADAMTS8 have also been identified for containing the antiangiogenic domain (TSR1) of thrombospondin. Both these proteins inhibited endothelial cell proliferation and suppressed growth factor induced vascularization of various assays [76]. Luque et al., elaborated on the function of ADAMTS1 to inhibit angiogenesis by decreasing VEGF. They found that ADAMTS1 binds VEGF which ultimately interferes with its ability to interact with its receptor VEGFR2, as was evident from lack of phosphorylation [77]. 

The ECM protein SPARC is a multifunctional matricellular glycoprotein that has been evaluated in various cancers but its role as either a tumor promoter or inhibitor has been controversial (reviewed by [78]). However, with respect to its effect on blood vessels, SPARC has been reported to be an antiangiogenic factor [79]. In this study, over-expression of SPARC in a glioma cell line resulted in a decrease in vascularity of xenograft tumors. It was also determined that the antiangiogenic effects of SPARC were associated with reduced tumor levels of VEGF [80]. SPARC also directly inhibits endothelial cell binding to the extracellular matrix by modulating extracellular calcium levels, effectively inhibiting blood vessel migration through the tumor stroma [81]. Other studies have shown that SPARC interferes with the growth promoting effects of VEGF on endothelial cells [82], and promotes the assembly of tightly organized stroma that does not permit blood vessel formation or tumor progression [81].

Lastly is a group of ECM proteins classified as endogenous inhibitors of angiogenesis which include various antiangiogenic peptides, hormone metabolites and modulators of apoptosis [83]. Therapeutic drugs have been developed based on matrix derived and nonmatrix-derived endogenous angiogenic inhibitors [83, 84]. These inhibitors can be classified based on whether they have solely angiogenic actions or whether they have functions in addition to angiogenic actions [84]. Of these, thrombospondin-1 was the first protein recognized as an endogenous inhibitor of angiogenesis [85] and has since become a popular target for the treatment of various cancers. The role of TSP-1 as an antiangiogenic protein will be extensively reviewed. 

Another class of proteins which have recently gained recognition for their potential role in tumor angiogenesis are the bone morphogenic proteins (BMPs). BMPs belong to the transforming growth factor beta (TGFb) superfamily of proteins and to date, more than 20 members have been identified [86]. An angiogenic role of the BMPs has been suggested due to a number of BMP mutations found in various vascular diseases and abnormal angiogenesis that occurs when BMP signalling is disrupted (reviewed by [87]). One of the mechanisms by which the BMPs have been reported to influence angiogenesis is by stimulating the secretion of proangiogenic growth factors such as VEGF [88]. BMP expression is associated with promoting tube formation and endothelial cell migration, whereas these activities are inhibited when BMP function is impaired [89]. Although BMPs are thought to stimulate angiogenesis, there have also been reports that demonstrate an antiangiogenic role [90, 91]. 

Aside from a direct role on blood vessel formation, recent studies have implicated interactions between BMPs and proteins of the ECM which can ultimately affect tumor growth and development. ECM proteins have been reported to interfere with BMP signalling by altering the bioavailability of TGFb (reviewed by [92]). BMPs are also thought to play a critical role in metastatic processes. In a model of prostate cancer, BMP-7 was shown to be highly expressed in bone and soft tissue metastases compared to the primary tumor and subcutaneous tumors formed from prostate adenocarcinoma cells overexpressing BMP-7 had a significantly reduced tumor volume compared to those with normal expression [93]. Similar results were reported in a model of lung carcinoma [94] and these results illustrate the relevance of the tissue microenvironment when studying how BMPs affect tumor growth. It is thought that tumor cells secrete BMPs which creates an environment that promotes tumor cell growth and metastasis [95]. Studies have shown that BMPs can contribute to decreased expression of various MMPs [96–98]. A reduction in MMP expression would permit metastatic cells to colonize and propagate in the tissue. The role of BMPs in primary and secondary tumor formation is not completely clear, however. Some evidence points to the need for BMP inhibition before metastatic spread can occur and it may be the case that the effects of members of the BMP family may be context-specific. In an ovarian cancer model, it was shown that overexpression of the BMP receptor, ALK3 decreases adhesion of epithelial ovarian cancer cells *in vitro *reduces formation of intraperitoneal tumors and ascites fluid *in vivo* [99]. Further understanding of the role which BMPs play in the ECM and with tumor angiogenesis will benefit therapeutic studies which target angiogenesis, tumour growth, and metastatic spread of disease. 

## 5. TSP Family of Proteins

Thrombospondin was originally identified as thrombin-sensitive protein by Baenziger et al., in the early 1970s. It was later realized that the protein was a subunit of a larger protein released from *α* granules of platelets in response to activation by thrombin. The native protein was officially named thrombospondin (TSP) [100, 101]. TSPs belong to a family of multifunctional glycoproteins that have a high affinity for matrix molecules, plasma proteins, ions, and various cell surfaces. They are capable of binding to heparin [102–104], fibronectin [105, 106], fibrinogen [106–108], plasminogen [109], histidine-rich glycoprotein [110], type IV collagen [111], and calcium [112, 113]. (For an extensive list of macromolecules that interacts with TSP see [114]. TSP is also capable of associating with various cell types and their corresponding extracellular matrices [115–119]. Combined, these diverse interactions allow TSP to be involved in cell-to-cell and cell-to-matrix communications (reviewed by [120]). 

TSP is a 450 kDA protein which is composed of three 150 kDA disulfide-linked polypeptide chains [101, 121–125]. Each subunit of the trimer consists of multiple domains: an N-terminal globular domain, a region homologous to procollagen, three types of repeated sequence motifs (type 1, type 2, and type 3 repeats) and a C-terminal globular domain [126]. There are five family members, TSP-1, -2, -3, -4 and -5 [127–129]. The TSP family can be divided into two subgroups on the basis of their oligomerization and molecular architecture. TSP-1 and -2 are trimers that have the same set of structural domains and belong to subgroup A. They are members of the thrombospondin type-1 repeat (TSR) supergene family whereas the remaining members of the family lack the TSR and the procollagen domain, are pentamers and are part of subgroup B [130]. 

## 6. Thrombospondin-1 (TSP-1) and Tumor Angiogenesis

Thrombospondin-1 (TSP-1) was the first of the five members to be identified as a major component of blood platelets. Since its discovery, TSP-1 has been implicated in the regulation of cell growth and proliferation [131, 132], cell motility [85, 133, 134], cytoskeletal organization [135, 136], inflammatory responses [137, 138], development and differentiation of various cell types [139], regulation of angiogenesis during wound healing [140], and tumorigenesis [141] (reviewed by [114]). 

 Due to the complex structure of TSP-1, there are multiple receptor binding domains located throughout the peptide that are capable of various functions [126]. These receptors include, low density lipoprotein receptor-related protein (LRP), proteoglycans and sulfatides, CD36, integrins, integrin-associated protein (IAP), and an unidentified receptor located in the C-terminus [142]. Many membrane proteins can also act as receptors for TSP-1 and activate downstream signalling pathways [143]. 

Many human tumor cell lines express relatively low levels of TSP-1 compared to normal or benign lines. It has also been observed that cell lines with low metastatic potential express higher levels of TSP-1 compared to metastatic lines [144, 145]. This has further been validated in experiments where TSP-1 transfection into human cancer cell lines inhibited primary tumor formation [145, 146] and decreased metastasis *in vivo *[145]. These results propose an inverse correlation between TSP-1 expression and tumor aggressiveness, whereby malignant progression is associated with reduced levels of TSP-1 in certain cancers. 

Overexpression of TSP-1 in cancer cell lines has been shown to suppress tumor formation by targeting the vasculature [145–150]. Studies involving introduction of TSP-1 into cell lines derived from gliobastoma multiforme induced the angiogenic switch to an antiangiogenic phenotype. Angiogenesis was measured by *in vitro *endothelial cell migration and *in vivo* corneal neovascularization assays [148]. Other *in vivo* studies have manipulated TSP-1 via transfection into human cancer cells lines and subsequent injection into nude mice. Angiogenesis is typically assessed based on microvessel density (MVD) which utilizes endothelial cell-specific markers, such as cluster of differentiation 31 (CD31) in order to perform vessel staining and counts. A decrease in MVD was observed in primary tumors that formed from the TSP-1 transfectant cell lines [145]. TSP-1 overexpression experiments in a model of human squamous cell carcinoma revealed consistent results in that tumor growth, vessel number, and size were drastically decreased. Histological examination demonstrated that tumors derived from TSP-1 stable transfected cells exhibited extensive areas of tumor cell necrosis which might have been due to the antiangiogenic effects of TSP-1 on tumor vasculature [149]. 

Clinical studies of patients with bladder, papillary thyroid and epithelial ovarian cancer have investigated levels of TSP-1 and correlated it with angiogenesis. This relationship was established based on a significant association between TSP-1 expression and MVD count. Tumors from patients that expressed high levels of TSP-1 had low MVD counts and were therefore more likely to exhibit a decrease in angiogenesis compared to control tissue. The inverse has also been documented; a decrease in TSP-1 expression was accompanied with high MVD counts which may contribute to an angiogenic phenotype [151–155]. 

Most studies have attempted to relate vascularity with expression of TSP-1 by probing tumor tissue with endothelial cell specific markers. Few studies have evaluated the expression of TSP-1 in hypo- compared to hypervascular carcinomas. There are however various human carcinomas with a varying degree of vascularization and have been used to determine how diminished vascularity relates to expression of TSP-1. It was found that a hypovascularized human carcinoma had increased levels of TSP-1 [156]. These results defend the inverse relationship that exists between the degree of vascularization and TSP-1 expression. They also support TSP-1 as an antiangiogenic protein that regulates tumorigenesis. 

TSP-1 expression has also been a predictor of tumor recurrence and overall survival. In clinical studies of patients with invasive bladder cancer and papillary thyroid carcinoma, low TSP-1 expression, as determined by immunohistochemistry, was associated with an increased probability of disease recurrence and decreased overall survival [151, 154]. Clinical studies of invasive epithelial and cervical cancer have revealed that TSP-1 expression is a valuable prognostic factor [155, 157]. In another study of invasive epithelial ovarian cancer, the majority of cases expressed high levels of TSP-1 which was associated with a higher survival rate compared to cases where tumors expressed lower levels of TSP-1 [152]. The 5-year survival rate of oral squamous cell carcinoma patients has also been shown to be significantly higher in tumors that express high levels of TSP-1 [153]. Based on this evidence, it is likely that TSP-1 possesses a tumor inhibitory function in some cancers and it may be a useful tool to assess prognosis.

## 7. Antiangiogenic Compounds and Vessel Normalization

The concept of antiangiogenic therapy for the treatment of various cancers was postulated in 1971 by Judah Folkman. It was hypothesized that solid tumor growth depends on angiogenesis in order to grow beyond 1-2 mm^3^. Therefore, it was thought that angiogenic inhibitors might be a potential therapeutic target; by blocking angiogenesis, tumor dormancy could be initiated [7, 158]. In 1996, Teicher proposed that antiangiogenic therapy would be most effective if used in combination with chemotherapy. The rationale was that the combinatorial effects would diminish the tumor cells as well as the endothelial cells associated with the tumor [159]. A hallmark of tumor angiogenesis is that blood vessels are formed so rapidly that they often become disorganized, torturous, and as a result have reduced functional capacity [160]. These immature blood vessels typically lack pericyte coverage, which may render them more vulnerable to apoptotic signals [161]. In 2001, Jain proposed the idea of tumor vasculature normalization as the product of antiangiogenic treatment. Because of the abnormal anatomy of tumor vessels perfusion is restricted, resulting in areas of tumor hypoxia and necrosis. This reduced blood flow to the tumor impairs the delivery of cytotoxic chemotherapeutic agents to the tumor interior, inhibiting their effectiveness, facilitating drug resistance and tumor regrowth [162, 163]. Anti-angiogenic therapy designed to target this abnormal, immature vasculature could effectively prune back vessels and increase blood flow, nutrient delivery, and waste removal. Combining vessel normalization with chemotherapy would provide better tissue perfusion of cytotoxic agents which induce apoptosis of the tumor [164]. It has since been reported that agents that inhibit proangiogenic factors alter the tumor vasculature and increase the delivery of chemotherapeutics when used in combination [165]. Recent studies have utilized the antiangiogenic peptide, TSP-1 for the treatment of various cancers and have found that the compounds are capable of normalizing tumor vasculature [166]. We have shown that TSP-1 directly inhibits VEGF and reduces its availability to ovarian cells [167]. In addition, we have shown that treatment with the TSP-1 mimetic peptide ABT-510 significantly reduces ovarian tumor volume and vascularity [168]. Importantly, treatment with ABT-510 decreased overall blood vessel density, but increased the proportion of mature, pericyte-covered blood vessels and decreased tumor tissue hypoxia. 

If normalized vessels increase the uptake of chemotherapeutic agents they may allow the drugs to be administered at lower doses, which would minimize their many deleterious side effects. The benefits are also supported by the fact that if the tumor vasculature is normalized instead of completely diminished, the tumor will not undergo hypoxia which is the major activator of VEGF, a potent proangiogenic factor. 

## 8. The Thrombospondins and Other EMC Proteins

The TSPs are also known to directly interact with other ECM proteins in their regulation of tumor progression and tumor angiogenesis. The Type 1 repeats of the TSP-1 and -2 genes inhibit MMP activity by preventing activation of the MMP-2 and -9 zymogens [169]. Conversely, others have reported that TSP-1 increases MMP-9 activity and tumor cell invasion [170], suggesting that the interaction between matricellular proteins may be context specific. TSP-1 null mice have reduced expression of TGF*β*, lower collagen content and delayed wound closure [171]. We also discovered that the ovaries of TSP-1 null mice were hypervascularized, with increased expression of VEGF [5]. *In vivo*, TSP-1 binds to a number of matrix glycosaminoglycans including heparan sulfate [172] chondroitin sulfate [173] and binds to members of the syndecan family, versican, and cerbroglycan [172–174] and these proteoglycans are thought to be important mediators of TSP-1. Aside from MMPs, TSP-1 is also known to inhibit the activity of plasmin, urokinase plasminogen activator, and elastin [175, 176], which are all key components of the extracellular matrix and are important in facilitating vessel invasion into the stroma. Aside from direct effects on VEGF expression and endothelial cells, TSP-1 appears to have potent antiangiogenic effects through its interaction with the extracellular matrix and on a host of matricellular proteins.

## 9. Conclusion

**Figure 1:**
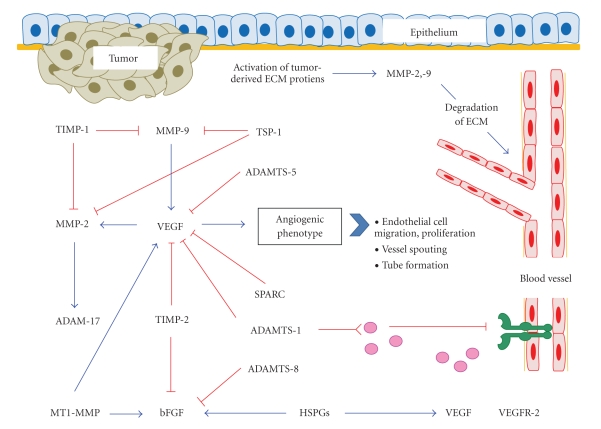
Summary of various extracellular matrix (ECM) proteins that are involved in tumour angiogenesis. Activation of tumor-derived ECM proteins permits communication between the tumor and its surrounding microenvironment. Regulating of angiogenesis can be directly through MMP activation and degradation of the ECM or through and indirect mechanism. This involves interactions between various ECM proteins and pro or antiangiogenic growth factors in order to alter angiogenesis. Ultimately, expression of the proangiogenic growth factor can influence the angiogenic phenotype and determine whether vascular sprouting occurs in order to provide the tumor with the necessary nutrients to survive.

Solid tumors exhibit significant structural complexity and progression of the disease is regulated by a host of different factors. This paper focused on the extracellular matrix as a major contributor to tumorigenesis. Once cells have undergone transformation and initiated the formation of a tumor, they must interact with the surrounding environment in order for tumor progression to occur [1]. This interaction activates tumor-derived ECM proteins which can have multiple effects on the tumor stroma in order to promote angiogenesis, a process which is essential for tumor growth [6]. The involvement of the ECM in tumor angiogenesis includes degradation of the basement membrane, matrix remodelling, and cell migration and invasion [32]. In this paper, we focused on ECM proteins that have both direct and indirect roles on the regulation of angiogenesis. Many of the ECM proteins appear to affect angiogenesis by altering expression of proangiogenic growth factors such as VEGF and bFGF. Other ECM proteins such as SPARC, ADAMs, and ADAMTs have also been investigated for their role in angiogenesis. These proteins have been shown to have direct antiangiogenic properties through their ability to inhibit proangiogenic growth factors. Lastly, we reported on the role of TSP-1 as it has been extensively studied with respect to tumor angiogenesis. It has been well documented that *in vitro*, TSP-1 decreases endothelial cell migration and invasion and decreases tumor vasculature *in vivo*. The involvement of ECM proteins in tumour angiogenesis is summarized in Figure 1. The potent antiangiogenic effects of TSP-1 have led to the development of mimetic peptides that have shown significant antiangiogenic and antitumorigenic effects *in vivo*. This review demonstrates the necessity for investigation of the microenvironment of the tumor and also supports the development of various therapeutics which can target ECM proteins.
